# Measuring a Broad Spectrum of eHealth Skills in the Web 3.0 Context Using an eHealth Literacy Scale: Development and Validation Study

**DOI:** 10.2196/31627

**Published:** 2021-09-23

**Authors:** Hua-Xuan Liu, Bik-Chu Chow, Wei Liang, Holger Hassel, YaJun Wendy Huang

**Affiliations:** 1 Department of Sport, Physical Education and Health Hong Kong Baptist University Hong Kong China (Hong Kong); 2 Center for Health and Exercise Science Research Hong Kong Baptist University Hong Kong China (Hong Kong); 3 Institute of Applied Health Sciences Coburg University of Applied Science and Arts Coburg Germany

**Keywords:** eHealth literacy, scale development, validation, college students

## Abstract

**Background:**

eHealth literacy (EHL) refers to a variety of capabilities that enable individuals to obtain health information from electronic resources and apply it to solve health problems. With the digitization of health care and the wide availability of health apps, a more diverse range of eHealth skills is required to properly use such health facilities. Existing EHL measurements focus mainly on the skill of obtaining health information (Web 1.0), whereas skills for web-based interactions (Web 2.0) and self-managing health data and applying information (Web 3.0) have not been well measured.

**Objective:**

This study aims to develop an EHL scale (eHLS) termed eHLS-Web3.0 comprising a comprehensive spectrum of Web 1.0, 2.0, and 3.0 skills to measure EHL, and evaluate its validity and reliability along with the measurement invariance among college students.

**Methods:**

In study 1, 421 Chinese college students (mean age 20.5, SD 1.4 years; 51.8% female) and 8 health experts (mean age 38.3, SD 5.9 years; 87.5% female) were involved to develop the eHLS-Web3.0. The scale development included three steps: item pool generation, content validation, and exploratory factor analysis. In study 2, 741 college students (mean age 21.3, SD 1.4 years; 52.2% female) were recruited from 4 Chinese cities to validate the newly developed eHLS-Web3.0. The construct validity, convergent validity, concurrent validity, internal consistency reliability, test-retest reliability, and measurement invariance across genders, majors, and regions were examined by a series of statistical analyses, including confirmatory factor analysis (CFA) and multigroup CFAs using SPSS and Mplus software packages.

**Results:**

Based on the item pool of 374 statements collected during the conceptual development, 24 items (4-10 items per subscale) were generated and adjusted after cognitive testing and content validity examination. Through exploratory factor analysis, a 3-factor eHLS-Web3.0 was finally developed, and it included acquisition (8 items), verification (6 items), and application (10 items). In study 2, CFAs supported the construct validity of the 24-item 3D eHLS-Web3.0 (χ^2^_244_=903.076, χ^2^_244_=3.701, comparative fit index=0.924, Tucker-Lewis index=0.914, root mean square error of approximation [RMSEA]=0.06, and standardized root mean residual [SRMR]=0.051). The average variance extracted (AVE) value of 0.58 and high correlation between eHLS-Web3.0 subscales and the eHealth Literacy Scale (*r*=0.725-0.880, *P*<.001) indicated the convergent validity and concurrent validity of the eHLS-Web3.0. The results also indicated satisfactory internal consistency reliability (*α*=.976, *ρ*=0.934-0.956) and test-retest reliability (*r*=0.858, *P*<.001) of the scale. Multigroup CFA demonstrated the 24-item eHLS-Web3.0 to be invariant at all configural, metric, strength, and structural levels across genders (female and male), majors (sport-related, medical, and general), and regions (Yinchuan, Kunming, Xiamen, and Beijing).

**Conclusions:**

The 24-item 3D eHLS-Web3.0 proved to be a reliable and valid measurement tool for EHL in the Web 3.0 context among Chinese college students.

## Introduction

With the digitization of health care and the wide availability of health applications, personal eHealth has become important for health management. The World Health Organization defines eHealth as “the use of information and communication technology for health” [1], and researchers have called for a broader understanding of the capabilities and skills required for individuals to use and benefit from eHealth services [2]. In 2006, Norman and Skinner labeled this ability as “eHealth literacy” (EHL) [3].

The definition of EHL was grounded in the health promotion theory, which referred to EHL as the “the ability to seek, find, understand, and appraise health information from electronic resources and apply that knowledge to solve a health problem or make a health-related decision” [3]. Based on this concept, an 8-item measurement tool named the eHealth Literacy Scale (eHEALS) was developed. The eHEALS is the most well-known and widely accepted tool for measuring EHL. Till 2015, there have been 45 studies that used the eHEALS, and it is the only tool used to measure EHL in more than 1 study [4].

However, with advances in technology, some people identified the need for updating the content of EHL to better fit it with the new internet environment. They also doubted on how accurately the eHEALS measured the use of new technologies to find and evaluate health information [5]. In 2011, the first critique of eHEALS was made, finding a weak correlation between the scale and eHealth behaviors beyond web-based information-researching skills, suggesting the need to revise the tool [6]. These observations were reasonable. According to the most widely accepted generation division of internet evolution, the current internet environment has gone through three stages. The first one, Web 1.0, refers to the read-only web, whereas Web 2.0 refers to a read-write mode, providing a *“*participative social web*”* with greater collaboration and interactivity between consumers, programmers, service providers, and organizations. Web 3.0 is the current environment, the so-called “semantic web,” accessible in a “read-write-execute” mode, providing digital, personal, and intelligent services [7-9]. The eHEALS was developed 15 years ago for measuring the abilities related to page views; it is necessary to enrich and update it for better scaling of current eHealth usage [4,5].

To fill this gap, other measures of EHL have been developed. For example, van der Vaart et al [6] designed 9 assignments to test the “actual performance of eHealth literacy.” However, those assignments were more for testing health-related internet skills rather than actual EHL. Moreover, the practical test approach would limit the number of participants. In 2017, another tool called the Digital Health Literacy Instrument was developed [10], which included 21 self-assessed items supplemented with 7 performance-related tasks that focused on handling digital information. They were mainly related to navigating the internet and messaging health professionals. The assignments developed by van der Vaart et al [6,10] were not the only ones to assess individuals’ EHL. The Readiness Self-Assessment-Health Professions version [11] also measured participants’ perceived ability and their actual ability to obtain and evaluate eHealth information. It is worthy to mention that the developers of the Readiness Self-Assessment-Health Professions version believed that the readiness to use internet-based resources should be seen as a component of EHL. The Patient Readiness to Engage in Health Internet Technology [12] measures readiness as well. It was developed based upon the groundwork of eHEALS but expanded the understanding of EHL to include two meta-factors: facilitators and barriers. Beyond task performance and readiness evaluations, several other measurements focused on evaluating people’s Web 2.0 skills, specifically their literacy on web-based social activities. One of them was the 19-item electronic Health Literacy Scale (eHLS) [13]. It examined the behavioral, communicational, and attitudinal components of health literacy among eHealth information seekers, which expanded the conceptualization beyond the traditional document-based measures to include interactive and communicative aspects of literacy. Another scale for Web 2.0 is the eHealth Literacy Scale (eHEALS) [14], suggesting that the underlying social structure affects an individual’s health status, computer literacy, intrinsic interest in health, and perceived ability to use the internet for health purposes. In addition, there were two other measurements that provided a different understanding of EHL. The eHealth Literacy Questionnaire (eHLQ) [15] comprehending EHL under a user-need perspective characterizing eHealth users paid attention to eHealth users’ understanding, attitudes, and motives. The eHealth Literacy Assessment Toolkit (eHLA) [16] was a mix of existing and newly developed scales that viewed EHL as a combination of health literacy, computer and digital literacy, and information literacy. A recent study translated the eHEALS [3] into Chinese and examined its psychometric properties among the Chinese population [17]. However, the referenced study has not measured individuals’ EHL in the context of Web 2.0 and 3.0, and the stability (eg, test-retest reliability and measurement invariance) of the EHL scale has not been examined.

Although previous studies have provided new ways to assess EHL with some of them scaling web communication abilities, all the tools mentioned above have not yet measured the skills of Web 3.0 technologies. Insufficient knowledge existed in the newly required competencies of EHL (eg, capacity for using mobile services, sense of information safety, and abilities of screening, communicating, and sharing). Furthermore, except for eHEALS, these scales have not been used by other researchers, which means there is still no well-developed scale for evaluating individuals’ EHL worldwide.

The COVID-19 pandemic led to a decrease in interpersonal social activities, but increased individuals’ EHL [18,19]. According to the annual report provided by a famous clinical virtual community in China publishing health care–related and clinical insights and findings [19], the visitor volume increased by 4.6 times compared to what it was earlier. Predictably, increased use of health information technology would change people’s health care styles, shifting partially from offline to internet-based services. In such an environment, it is essential to develop a new instrument and provide a valid tool to measure current EHL levels. As college students are the major internet users and frequently turn to it for eHealth information [20], the current research aimed to develop a scale to measure EHL in the Web 3.0 context (eHLS-Web3.0) and examine its reliability and validity among Chinese college students. Accordingly, two studies were implemented; the first study aimed to develop an eHealth literacy scale (eHLS-Web3.0), including three steps: item pool generation, content validation, and exploratory factor analysis (EFA); the second study aimed to examine the validity (construct, convergent, and concurrent), reliability (internal consistency and test-retest reliability) and measurement invariance across genders (female and male), majors (sport-related, medical, and general), and regions (Yinchuan, Kunming, Xiamen, and Beijing) in Chinese college students. The current research was approved by the Research Ethics Committee of Hong Kong Baptist University.

## Methods

### Study 1

#### Participants

##### Step 1: Item Pool Generation

This step involved 28 college students, including 18 interviewees for item pool generation and 10 students for readability and cultural sensitivity examination. Snowball sampling [21] was applied in 2019 (before the COVID-19 pandemic) to recruit interviewees from three different Chinese cities (Beijing, Wuhan, and Putian). These three cities were selected based on the cultural geographic (north, middle, and south) and economic statuses (high, medium, and low) of these Chinese cities [22-24].

All eligible participants met the following inclusion criteria: (1) experience in using eHealth websites and tools, (2) having sufficient Chinese language skills, and (3) willingly consent to participate in the interview. Regarding the sample size, although there is no definite criterion for qualitative research, the number of participants should be in accordance with the creed of theoretical saturation, which means no new or relevant data emerge [25]. To achieve theoretical saturation, based on the “rule of thumb,” the suggested number of participants for interview studies is approximately 12 to 15 [26]. In the current study, 18 Chinese college students participated in the semistructured interviews via telephone; they included 3 males and 3 females from sports, medical, and ordinary nonhealth-related majors. All the telephonic interviews were conducted in Chinese (Mandarin) language and recorded digitally with the interviewees’ consent. An initial item pool was built based on the interview data and the existing literature. Next, 10 college students were randomly recruited to examine accessibility (eg, readability) and cultural sensitivity of those candidate items, and the participants’ ability to complete the self-administered survey. An informed consent form was delivered to each student before data collection.

##### Step 2: Content Validation

Based on the guideline proposed by Lynn [27], 8 Chinese health experts were invited to validate the content of the selected items [27]. The selection of the health experts was based on the following criteria: (1) published at least one paper related to health literacy or eHealth, (2) have sufficient Chinese language proficiency, and (3) confirmed their willingness and consent for participating in the interview.

##### Step 3: EFA Process

The participants involved in this step constituted an independent sample with the recruitment criteria similar to those in the interviewees of step 1, except that the target cities for participant recruitment were changed. This is because step 3 was performed after the outbreak of COVID-19. The previous target city Wuhan was avoided to enhance the generalizability of the new scale. In step 3, four different representative Chinese cities were chosen, including Beijing, Xiamen, Kunming, and Yinchuan. These four cities were selected based on their geographic locations (north, southeast, southwest, and northwest), political status (country capital, provincial capital, and prefecture-level city), and economic status (high, medium, and low) [22-24]. Furthermore, these four cities were chosen considering the issues of “convenience and feasibility” [28].

The questionnaire was distributed via a survey distribution website, starting with prior informed consent, followed by the main content of the survey. The participation was voluntary, and all participants were allowed to withdraw from the study at any time. University lecturers from the target cities helped distribute the link to the questionnaire in the classes. Based on the suggestion of Bryman [25,29,30], at least 240 observations were required for EFA (1:10 item-to-response ratio) in the current study. Finally, 393 students responded to the questionnaire and provided information regarding their age, gender, major, and experience of using health-related electronic tools or websites, which led to an adequate sample size. It took approximately 5 to 10 minutes for the participants to complete the questionnaire. The characteristics of the participants in study 1 are presented in Table 1.

**Table 1:** Characteristics of participants in study 1.

Demographic information		Values
**Interviewees (n=18)**		
	Age (years), mean (SD*)*	22.1 (1.02)
	Age range	21-25
	Gender (female), n (%)	9 (50)
**Readability examiners (n=10)**		
	Age (years), mean (SD)	20.3 (0.95)
	Age range	19-22
	Gender (female), n (%)	7 (70)
**Experts for content validation (n=8)**		
	Age (years), mean (SD)	38.3 (5.92)
	Age range	30-46
	Gender (female), n (%)	7 (87.5)
**Sample for EFA^a^ (n=393)**		
	Age (years), mean (SD)	20.5 (1.36)
	Age range	18-25
	Gender (female), n (%)	202 (51.4)

^a^EFA: exploratory factor analysis.

#### Procedure

##### Step 1: Item Pool Generation

There were three types of sources for forming the item pool. The first comprised well-established EHL instruments (eg, eHEALS, eHLQ, and e-HLS). The feasible items were selected and adapted to form the initial item pool. Second, items from well-known health or computer literacy scales were deliberately selected because they were measuring important elements of EHL. Third, items were generated from the previous interview results. For the first and second types of sources, some items were developed by western scholars. Those items were translated by two PhD students specializing in health promotion, and then back-translated by a senior Chinese English teacher unfamiliar with eHealth-literacy–related indices. Subsequently, 10 college students helped assess the comprehensibility, clarity, and length of the items via a dichotomous scale (applicable vs inapplicable). The inapplicable items were deleted or refined.

##### Step 2: Content Validation

The generated item pool was sent to the panel of experts. The experts assessed the relevance of each item to the understanding of EHL, using a 4-point Likert scale ranging from 1 (irrelevant) to 4 (extremely relevant). They were asked to provide ratings and suggestions for alternative items. The ratings were used to calculate the content validity index [27] of each item (I-CVI). The I-CVI was calculated by dividing the number of judges providing a rating above 3 by the total number of judges. The acceptable I-CVI score had to be above 0.83 [27,31]. The invalid items were either eliminated or revised based on the experts’ suggestions.

##### Step 3: EFA Process

The items generated in step 2 were placed in the format of a 5-point Likert scale, ranging from 1 (strongly disagree) to 5 (strongly agree). The distribution pattern of the items was examined. The ones with non-normal distributions were eliminated. EFA was performed for the remaining items. Principal component factoring with direct oblimin rotation was performed. Factor extraction was based on an eigenvalue higher than 1 and a confirmatory inspection of the scree plot. Items with primary factor loadings of 0.4 and above were considered interpretable [32,33]. Items with cross-loading were deleted, which refers to those having a second highest factor loading of 0.3 or higher or having a small gap (less than 0.2) between the primary and secondary loadings [32,33]. Bivariate correlations between the subscales were also tested.

### Study 2

#### Participants

The participants were independent samples with the recruitment criteria similar to those used in the EFA step. Considering the item-to-response ratios of at least 1:10 [29] and the recommendation for a minimum sample size of 200 in confirmatory factor analysis (CFA) [34], 741 college students were invited to participate in the current study. After 1.5 months, a follow-up retest was distributed to the same group of people, and 306 of them responded. A summary of the CFA participants is presented in Table 2.

**Table 2:** Sample characteristics for confirmatory factor analysis (N=741).

Demographic information		Value
Age (years), mean (SD)		21.3 (1.39)
Age range		17-25
Gender (female), n (%)		387 (52.2)
**Major**		
	Medical, n (%)	41 (5.5)
	Sport, n (%)	128 (17.3)
	Nonhealth-related, n (%)	572 (77.2)
**Region**		
	Beijing, n (%)	124 (16.7)
	Xiamen, n (%)	287 (38.7)
	Kunming, n (%)	216 (29.1)
	Yinchuan, n (%)	114 (15.4)

#### Measures

The eHLS-Web3.0 instrument was developed in study 1, consisting of 24 items classified based on 3 factors: acquisition (8 items), verification (6 items), and application (10 items). The answers were indicated on a 5-point Likert scale ranging from 1 (strongly disagree) to 5 (strongly agree). The internal consistency (Cronbach α) for the total scale was .971 and those for the subscales were 0.913-0.962 in Chinese college students.

The eHEALS was initially developed by Norman and Skinner [3] as the first and most widely used tool for measuring EHL. It is an 8-item unidimensional scale whose validation was performed in a population of adolescents (aged 13-21 years) from 14 secondary schools in a large Canadian city. The Chinese version of eHEALS was tested in 110 senior high school students by Guo et al [35]. The internal consistency (Cronbach α) for the total scale was .913, and the factor loading coefficients were between 0.692 and 0.869 [35] in Chinese senior high school students.

#### Procedure

The procedures for data collection were identical to those of the EFA step. Participants were selected from four target cities, Beijing, Xiamen, Kunming, and Yinchuan, by snowball sampling. The eHLS-Web3.0 was distributed to participants who were independent of those participating in the EFA step via the internet. University lecturers from the target cities independent of those in the EFA helped distribute the link to the questionnaire in the classes they taught; they consciously avoided the classes that had participants in the EFA step. Informed consent forms were delivered to the participants before the survey. It took approximately 10 minutes for the participants to finish the questionnaire. A retest was delivered to them 1.5 months later.

#### Data Analysis

To test the construct validity of the current scale, the items were analyzed through CFA with structural equation modeling using Mplus (version 7) [36]. After testing the distribution of the items in the measurement model, the maximum likelihood robust estimator was employed [37]. The chi-square statistic (χ^2^) was used to test the model’s overall goodness of fit [38]. Multiple model fit indices were examined further, including the comparative fit index [39] and Tucker-Lewis index [40], with a cutoff value of approximately 0.9 and above indicating a satisfactory fit between the CFI and TLI [41]; the standardized root mean residual (SRMR) [39,41] with values near 0.08 indicates adequate model fit; the root mean square error of approximation (RMSEA) and its 90% CI [42] indicate good fit at values less than 0.08. The standardized factor loadings and standardized residuals were examined. Items with factor loadings below 0.4 and large standardized residuals (≥2) were removed. The concurrent validity was examined by calculating the bivariate correlations between the subscale of the eHLS-Web3.0 and eHEALS using SPSS (version 24.0, SPSS Inc). The convergent validity was tested by examining the average variance extracted (AVE), for which values above 0.5 indicated an acceptable measurement. Internal consistency reliability was examined by analyzing the Cronbach α and the composite reliability coefficient (CR), for which the CR values above 0.7 [43] were considered acceptable. The test-retest reliability was measured by examining the bivariate correlation between the score of eHLS-Web3.0 determined in the CFA stage and the follow-up stage after 1.5 months. A multiple-group covariance structure analysis approach was employed to examine whether the measurement was invariant across genders, majors, and regions [44]. A ΔCFI value smaller than or equal to 0.01 between incrementally constrained models should be accepted as evidence of adequate fit [45].

## Results

### Study 1

#### Step 1: Item Pool Generation

**Figure 1:**
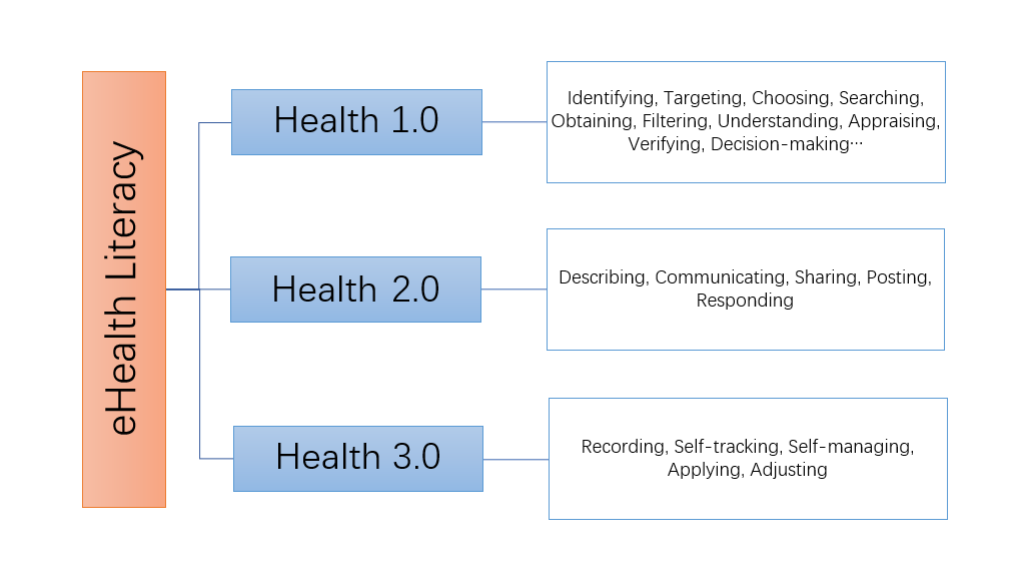
Conceptual framework of eHealth literacy.

Previous interviews yielded 374 tags and a conceptual framework for EHL, as shown in Figure 1.

The Health 1.0 part was consistent with existing research [3,4,6,10-12]; some Health 2.0 behaviors (describing, communicating, and sharing) [14,15] have also been mentioned by other researchers. In contrast, this study presents a first look at behaviors related to Web 3.0 and delves deeper into the social network services with respect to eHealth, finding that the application of information is currently much more complicated than before; other than researching on the internet or applying the information to make health-related decisions or solve problems, individuals could also create their own health data, use the information to communicate, respond (ie, giving suggestions and advice, and responding to help seekers), socialize with others, and share and post information (ie, forward helpful information or post their own health or fitness data).

Based on the abovementioned tags and existing well-established scales, 266 items were developed, taking the form of eHEALS (eg, I know how to find helpful health resources on the internet) in the Chinese language. Specifically, there were 114 items for Health 1.0 skills, 39 for Health 2.0 ones, 55 for Health 3.0 ones, 42 for readiness-related ones (mostly retrieved from earlier studies) [11,12], and 16 for health literacy ones (as per an earlier study) [10]. To ensure the relevance, readability, comprehensibility, and clarity of the scale to the targeted Chinese population, it was assessed by 10 college students who were conveniently selected. Suggestions were given by the participants to improve the comprehensibility of the preliminary pool. Among the items, 7 duplicated ones were discussed, 3 of them were deleted, and 4 of them were merged into 2 items. Next, 5 items were removed owing to unclear text. Further, 56 items were excluded because they were considered inapplicable for testing EHL by more than 3 students. After that, 200 candidate items were retained for the next step.

#### Step 2: Content Validation

Following the adjustments of the item pool, the candidate items were forwarded to 8 specialists in the health literacy and eHealth areas to review the content and assess its validity. Based on their quantitative ratings, the I-CVI was calculated for individual items. Items rated “1” or “2” indicating inapplicability according to two reviewers (I-CVI<0.83) were subsequently deleted. Additionally, suggestions regarding refinement of the scenario or item pool were elicited.

Based on the specialists’ quantitative feedback, 128 items were considered irrelevant and excluded. For the remaining 72 items, a discussion among specialists was arranged via a web-based group chat and videoconferencing for obtaining more detailed suggestions. Some mergers and adjustments were made to make the scale the clearest and the most easily understandable one. For instance, in the initial version, the abilities to choose proper forums and apps were measured separately following expert suggestions; these items were merged into one using the term “eHealth tools.” The questions on web-based celebrity and official accounts were also conflated. Some items testing attitudes for eHealth, especially the readiness-related ones, were considered not related to “ability” and eliminated. After the creation and refinement steps, the final number of items for field testing was 50.

#### Step 3: EFA Process

**Figure 2:**
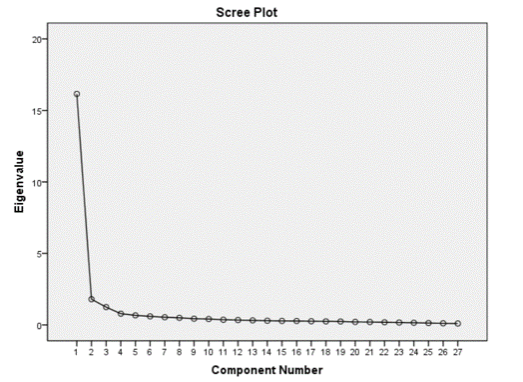
Confirmatory inspection of the scree plot.

The 50 refined items were randomized and administered to 393 college students via the internet. Descriptive statistical analysis was performed with SPSS (Version 20) examining the distribution pattern of each item. Items 2, 10, 37, and 50 were non-normally distributed (their kurtosis ranges were beyond –1 and 1). For the 46 remaining items, EFA was performed. The results of the Kaiser-Meyer-Olkin **(**KMO) and Bartlett tests performed during EFA showed that the KMO statistic for these items was 0.966, which is higher than 0.6, thus proving that the factor analysis model was appropriate and useful to analyze current data sets. Principal component factoring with direct oblimin rotation was used. Factor extraction was based on an eigenvalue higher than 1 and a confirmatory inspection of the scree plot shown in Figure 2. Items with primary factor loadings of 0.4 and above were considered interpretable. We excluded 5 items (#1, #11, #27, #28, and #36) with inadequate factor loadings were excluded. Subsequently, 13 other items were found to have a second highest factor loading of 0.3 or higher, and 4 other items (#22, #29, #33, and #41) had a small gap (less than 0.2) between the primary and secondary loadings. They were identified as items with cross-loading and were deleted as well. Therefore, 5 factors were extracted from initial factoring, and 3 strong factors were identified with the 24 items remaining, as shown in Table 3.

**Table 3:** Principal component analysis of the electronic Health Literacy Scale-Web3.0.

Item	Component		
	1	2	3
#3: I know which kind of eHealth tool I should choose to fit my health needs (ie, checking drug description, seeking health advice, or making weight loss plan).	—^a^	—	–0.817
#4: I can judge whether the eHealth tool is credible or not.	—	—	–0.659
#5: I will prefer to obtain the health information (ie, information about medical, sport or daily care) via offline channel.	—	—	0.880
#6: I know where to find useful health resources on the internet.	—	—	–0.424
#26: When communicating with others on the internet, I can articulate my health-related concerns clearly.	—	—	–0.530
#30: When replying to others’ health-related help-seeking on the internet, I can provide responsible response (which means my answer can neither mislead others, nor)	—	—	–0.547
#31: I can judge whether the health information available on the internet has a commercial interest (ie, the person providing the information is for the sale of a product).	—	—	–0.557
#35: When using the eHealth tools, I will protect the originality of the information (ie, never plagiarize others’ original content, report an offense to those infringing ones).	—	—	–0.550
#14: When searching the health information on the internet, I will check the credentials and affiliations of author.	—	–0.979	—
#15: When searching the health information on the internet, I will check who owns the website.	—	–0.938	—
#16: When searching the health information on the internet, I will check the date of its last update.	—	–0.864	—
#17: When searching the health information on the internet, I will check whether other print or web resources had confirmed this information.	—	–0.903	—
#18: I know how to verify the eHealth information from multiple sources.	—	–0.510	—
#20: Even if the health information I obtained is from someone I trust, I will still check it on the internet.	—	–0.444	—
#38: I know how to use the eHealth tools to record my health behaviors.	0.873	—	—
#39: I know how to make use of the records on the eHealth tools to provide reference for my daily health management.	0.852	—	—
#40: I know how to use the eHealth tools to track my health behaviors (ie, acquainting my exercise frequency or the change curve of body fat rate).	0.869	—	—
#42: I can continuously use a certain eHealth tool (ie, apps, intelligent body fat scale, fitness bracelet) for a long time.	0.919	—	—
#43: I can use the eHealth tools with a clear plan.	0.863	—	—
#44: I can adjust my frequency, strength, and usage pattern timely when using the eHealth tool according to the actual condition.	0.879	—	—
#45: I know how to use the eHealth tools to post and share my eHealth behaviors (ie, post my motion trails on health apps or moments on WeChat).	0.922	—	—
#46. I know how to use the sports functions on social network services (such as WeRun on WeChat) to interact with others (eg, thumb up, forward).	0.688	—	—
#47: I will target the advanced players I follow on the eHealth tools, learn from them, and catch up with them.	0.793	—	—
#48: I will try out some health-related suggestions available on the internet and control the risks (ie, get injured or take the wrong medicine).	0.811	—	—
Eigenvalue	14.439	1.658	1.114
Variance (%)	60.163	6.909	4.643
Cumulative (%)	60.163	67.072	71.715

^a^Not available.

Thus far, the current instrument has 24 items that were sorted based on 3 factors: acquisition (8 items), verification (6 items), and application (10 items). The total variance of this scale explained by these 3 factors was 71.715%. The internal consistency of each factor was also tested using the Cronbach α, as shown in Table 4. The result was satisfactory.

Bivariate correlations between the subscales were tested, and they are shown in Table 5. The moderate strength of the correlations indicated that the subscales were measuring related but distinct constructs.

**Table 4:** Reliability statistics.

Scale	Score, mean (SD*)*	Cronbach α
Scale in total	86.27 (14.5)	.971
Subscale of Factor 1	36.25 (7.5)	.962
Subscale of Factor 2	21.96 (7.5)	.934
Subscale of Factor 3	28.06 (4.0)	.913

**Table 5:** Correlations between the subscales.

			Subscale 1		Subscale 2		Subscale 3
**Subscale 1**							
	*r*	1		—^a^		—	
	*P* value	—		—		—	
**Subscale 2**							
	*r*	0.774		1		—	
	*P* value	<.001		—		—	
**Subscale 3**							
	*r*	0.783		0.726		1	
	*P* value	<.001		<.001		—	

^a^Not available.

It was surprising that positive and negative factor loadings existed simultaneously in the principal component analysis. Reviewing the scale showed that only item 5 was asked in a reverse manner, which might have confused participants and led to such a case. After discussion, the authors decided to refine item 5 as “I will obtain the health information (ie, medical, sport, or daily care information) on the internet.” In addition, a conditional statement “If needed, I can*…*” was added to items 18 and 19 in case some participants did not use eHealth tools frequently. As a result, the adjusted 24 items in 3 dimensions comprising 6, 8, or 10 items in each were developed to yield the new eHLS-Web 3.0 instrument (see Multimedia Appendix 1).

### Study 2

#### Construct Validity of eHLS-Web3.0

The primary CFA showed that the model did not fit the data very well, with fit indices of χ^2^_249_=1351.230, χ^2^_249_=5.427, CFI=0.873, TLI=0.860, RMSEA=0.077, and SRMR=0.056. According to the model modification indices, the residual errors of 5 items (#1, #15, #19, #21, and #23) were corrected. After modification, the eHLS-Web3.0 met the criteria for good model fit, with χ^2^_244_=903.076, χ^2^_244_=3.701, CFI=0.924, TLI=0.914, RMSEA=0.06, and SRMR=0.051. The factor loadings (see Multimedia Appendix 2) obtained using the 3-factor model were found to fit well with the data. The standardized loadings were all above 0.6, most of which were greater than 0.8. Correlations between the 3 factors were moderate to high.

#### Convergent and Concurrent Validities of eHLS-Web3.0

The AVE was calculated as 0.52 using the data collected in the EFA stage, showing an acceptable convergent validity. For the concurrent validity, the bivariate correlations between the subscale of the eHLS-Web3.0 and eHEALS were significant, showing a satisfactory result, as indicated in Table 6.

**Table 6:** Correlations between subscales of the electronic Health Literacy Scale-Web3.0 and eHealth Literacy Scale.

			eHLS-Web3.0^a^		Subscale 1		Subscale 2		Subscale 3
**eHEALS^b^**									
	*r*	0.893		—^c^		—		—	
	*P* value	<.001		—		—		—	
**Subscale 1**									
	*r*	0.880		—		—		—	
	*P* value	<.001		—		—		—	
**Subscale 2**									
	*r*	—		—		0.725		—	
	*P* value	—		—		<.001		—	
**Subscale 3**			—		—		—		—
	*r*	—		—		—		0.853	
	*P* value	—		—		—		<.001	

^a^eHLS-Web3.0: electronic Health Literacy Scale-Web3.0.

^b^eHEALS: eHealth Literacy Scale.

^c^Not applicable.

#### Internal Consistency and Test-Retest Reliabilities of eHLS-Web3.0

The Cronbach α, composite reliability coefficient, and the item-total correlations were calculated to examine the internal consistency reliability of the new instrument. The specific results are presented in Table 7, indicating satisfactory internal consistency reliability.

An acceptable test-retest reliability was achieved, showing that the eHLS-Web3.0 was relatively stable over time, as shown in Table 8.

**Table 7:** Internal consistency reliability statistics.

Statistic		Value
**Subscale 1**		
	Range of interitem correlations	0.549-0.832
	Minimum corrected item-total correlation	0.726
	Composite reliability coefficient	0.937
	Cronbach α	.950
**Subscale 2**		
	Range of interitem correlations	0.726-0.807
	Minimum corrected item-total correlation	0.828
	Composite reliability coefficient	0.934
	Cronbach α	.935
**Subscale 3**		
	Range of interitem correlations	0.511-0.898
	Minimum corrected item-total correlation	0.673
	Composite reliability coefficient	0.956
	Cronbach α	.958
**eHLS-Web3.0^a^**		
	Range of interitem correlations	0.470-0.898
	Minimum corrected item-total correlation	0.858
	Composite reliability coefficient	0.981
	Cronbach α	.976

^a^eHLS-Web3.0: electronic Health Literacy Scale-Web3.0.

**Table 8:** Correlations between test (T1) and retest (T2).

Test		T1		T2
**T1**				
	Mean (SD)	83.97 (14.67)	—^a^	
	*r*	—	—	
	*P* value	<.001	—	
**T2**				
	Mean (SD)	—	86.37 (15.35)	
	*r*	—	0.858	
	*P* value	—	<.001	

^a^Not applicable.

#### Measurement Invariance of eHLS-Web3.0

Multigroup CFAs were employed to examine whether the measurement was invariant across genders, majors, and regions. Results of the invariance analyses are provided in Tables 9-11.

For the gender invariance analysis presented in Table 9, it was found that the configural model (M0) showed satisfactory fit to the data. The matric model (M1) and strong model (M2) displayed satisfactory fit to the data, and their △CFI was less than 0.01; the △RMSEA was less than 0.01, and the △SRMR was less than 0.025. These indices supported the invariance of factor loadings and intercepts across genders. The strict model (M3) reflected acceptable fit to the data. Although the △SRMR in M3 showed poor fit, the other indices showed a satisfactory goodness of fit (△CFI and △RMSEA<0.01), indicating that the residual errors were equivalently invariant across genders.

For the major invariance analysis, the model fitness tests and comparisons were performed across all three samples, including general (nonhealth-related), sport, and medical major students. The configural model (M0) showed satisfactory fit to the data between general and sport as well as general and medical, whereas the configural model reflected a marginal-to-acceptable fit (CFI=0.849) between sport and medical majors. This is most likely because the medical group had a much smaller sample size than the sport group. As for the goodness of model fit, the matric model (M1), strong model (M2), and strict model (M3) displayed satisfactory and acceptable fit to the data between general and sport as well as general and medical, and marginal-to-acceptable fit to the data between sport and medical groups. The △CFI and △RMSEA of all these models were less than 0.01, and the △SRMR was less than 0.025. These indices supported the invariance of the factor loadings, intercepts, and residual errors across majors, as observed in Table 10.

**Table 9:** Invariance analysis of the electronic Health Literacy Scale-Web3.0 across genders (nmale=354 and nfemale=387).

Model	*χ*^2a^ (*df*)	CFI^b^	△CFI^c^	RMSEA^d^	△RMSEA^e^	SRMR^f^	△SRMR^g^
M0^h^	1215.588 (488)	0.918	—^i^	0.063	—	0.057	—
M1^j^	1264.312 (488)	0.915	–0.003	0.063	0	0.064	0.007
M2^k^	1328.711 (488)	0.91	–0.005	0.063	0	0.067	0.003
M3^l^	1368.771 (539)	0.906	–0.004	0.064	0.001	0.205	0.138

^a^χ^2^: chi-square ratio statistic.

^b^CFI: comparative fit index.

^c^△CFI: change in the comparative fit index.

^d^RMSEA: root mean square error of approximation.

^e^△RMSEA: change in the root mean square error of approximation.

^f^SRMR: standardized root mean residual.

^g^△SRMR: change in the standardized root mean residual.

^h^M0: baseline configural invariance model.

^i^Not available.

^j^M1: metric invariance model.

^k^M2: strong invariance model.

^l^M3: strict invariance model.

**Table 10:** Invariance analysis of the electronic Health Literacy Scale-Web3.0 across majors (ngeneral=572, nmedical=41, and nsport=128).

Model		*χ*^2a^ (*df*)	CFI^b^		△CFI^c^	RMSEA^d^		△RMSEA^e^		SRMR^f^	△SRMR^g^
**General and sport-related majors**											
	M0^h^	1240.042 (488)	0.922	—^i^		0.066	—		0.05		—
	M1^j^	1272.243 (509)	0.92	–0.002		0.065	–0.001		0.053		0.003
	M2^k^	1320.117 (533)	0.918	–0.002		0.065	0		0.061		0.008
	M3^l^	1326.536 (539)	0.918	0		0.065	0		0.059		–0.002
**General and medical majors**											
	M0	1354.433 (488)	0.912	—		0.076	—		0.056		—
	M1	1394.817 (509)	0.91	–0.002		0.075	–0.001		0.064		0.008
	M2	1448.693 (533)	0.907	–0.003		0.075	0		0.066		0.002
	M3	1451.485 (539)	0.907	0		0.074	–0.001		0.067		0.001
**Medical and sport-related majors**											
	M0	952.825 (488)	0.849	—		0.106	—		0.068		—
	M1	991.739 (509)	0.843	–0.006		0.106	0		0.092		0.024
	M2	1031.023 (533)	0.838	–0.005		0.105	–0.001		0.095		0.003
	M3	1035.032 (539)	0.839	0.001		0.104	–0.001		0.099		0.004

^a^χ^2^: chi-square ratio statistic.

^b^CFI: comparative fit index.

^c^△CFI: change in the comparative fit index.

^d^RMSEA: root mean square error of approximation.

^e^△RMSEA: change in the root mean square error of approximation.

^f^SRMR: standardized root mean residual.

^g^△SRMR: change in the standardized root mean residual.

^h^M0: baseline configural invariance model.

^i^Not applicable.

^j^M1: metric invariance model.

^k^M2: strong invariance model.

^l^M3: strict invariance model.

For the region invariance analysis, the model tests and comparisons were conducted across all the four groups of samples, including students from Kunming, Yinchuan, Xiamen, and Beijing. The configural model (M0) showed acceptable fit to the data across all the four regional samples. For the goodness of model fit, the matric model (M1) and strong model (M2) for every group displayed acceptable fit to the data. For every group, the △CFI and △RMSEA of the models were less than 0.01, and the △SRMR was less than 0.025. These indices supported the invariance of the factor loadings and intercepts across regions. The strict model (M3) for each group also reflected acceptable fit to the data. However, between Kunming and Yinchuan, Kunming and Xiamen, Yinchuan and Xiamen, and Xiamen and Beijing, the △SRMR of the strict model (M3) reflected a poor fit, as shown in Table 11. Despite this, the other indices showed a satisfactory goodness of fit (the △CFI and △RMSEA were less than 0.01), proving that the residual errors were equivalently invariant across the regions.

In summary, these findings supported the measurement invariance of the eHLS-Web3.0 model (factor loadings, intercepts, and residual errors) across genders, majors, and regions.

**Table 11:** Invariance analysis of the electronic Health Literacy Scale-Web3.0 across regions (nKunming=216, nYinchuan=114, nXiamen=287, and nBeijing=124).

Region			χ^2a^ (*df*)		CFI^b^		△CFI^c^		RMSEA^d^		△RMSEA^e^		SRMR^f^	△SRMR^g^
**Kunming and Yinchuan**														
	M0^h^	1045.16 (488)		0.875		—^i^		0.083		—		0.068		—
	M1^j^	1074.43 (509)		0.873		–0.002		0.082		–0.001		0.076		0.008
	M2^k^	1126.886 (533)		0.867		–0.006		0.082		0		0.082		0.006
	M3^l^	1137.122 (539)		0.866		–0.001		0.082		0		0.107		0.025
**Kunming and Xiamen**														
	M0	1127.636 (488)		0.908		—		0.072		—		0.058		—
	M1	1172.651 (509)		0.904		–0.004		0.072		0		0.071		0.013
	M2	1228.577 (533)		0.899		–0.005		0.072		0		0.079		0.008
	M3	1249.567 (539)		0.897		–0.002		0.072		0		0.182		0.103
**Kunming and Beijing**														
	M0	1023.786 (488)		0.879		—		0.08		—		0.074		—
	M1	1038.028 (509)		0.88		0.001		0.078		–0.002		0.078		0.004
	M2	1095.957 (533)		0.872		–0.008		0.079		0.001		0.083		0.005
	M3	1102.118 (539)		0.872		0		0.078		–0.001		0.098		0.015
**Yinchuan and Xiamen**														
	M0	1043.404 (488)		0.908		—		0.075		—		0.047		—
	M1	1078.271 (509)		0.906		–0.002		0.075		0		0.054		0.007
	M2	1118.863 (533)		0.903		–0.003		0.074		–0.001		0.055		0.001
	M3	1128.242 (539)		0.903		0		0.074		0		0.109		0.054
**Yinchuan and Beijing**														
	M0	917.12 (488)		0.874		—		0.086		—		0.067		—
	M1	936.311 (509)		0.875		0.001		0.084		–0.002		0.076		0.009
	M2	1008.445 (533)		0.861		–0.014		0.087		0.003		0.085		0.009
	M3	1024.654 (539)		0.858		–0.003		0.087		0		0.094		0.009
**Xiamen and Beijing**														
	M0	1026.47 (488)		0.913		—		0.073		—		0.054		—
	M1	1058.117 (509)		0.912		–0.001		0.072		–0.001		0.063		0.009
	M2	1126.022 (533)		0.904		–0.008		0.074		0.002		0.069		0.006
	M3	1137.64 (539)		0.904		0		0.074		0		0.117		0.048

^a^χ^2^: chi-square ratio statistic.

^b^CFI: comparative fit index.

^c^△CFI: change in the comparative fit index.

^d^RMSEA: root mean square error of approximation.

^e^△RMSEA: change in the root mean square error of approximation.

^f^SRMR: standardized root mean residual.

^g^△SRMR: change in the standardized root mean residual.

^h^M0: baseline configural invariance model.

^i^Not available.

^j^M1: metric invariance model.

^k^M2: strong invariance model.

^l^M3: strict invariance model.

## Discussion

### Principal Findings

The current study developed and tested a new measurement tool for EHL in Chinese with the Web 3.0 context named eHLS-Web3.0. A multistage program was applied, generating 24 items that represent the updated content of EHL in the current internet environment. In comparison with other eHealth models [3,5], the new instrument measures not only the skills for searching eHealth information (Health 1.0 skills) or communicating with service providers (Health 2.0 skills), but also the skills for building personal health data sets, self-tracking, and protecting privacy (Health 3.0 skills). Although the items were built upon the groundwork laid by the eHEALS [3], with the insights from the participants’ usage of the Web 3.0 technology, eHLS-Web3.0 went beyond the computer skill and media literacy components of the eHEALS. It provided a much deeper understanding of people’s interactions with different types of eHealth tools and the application of the information they obtained or created. Construction and validity testing in a broad range of target groups generated clear evidence of construct, convergent, and concurrent validities, as well as composite and internal consistency, and test-retest reliability. Measurement invariance was also found across genders, majors, and regions. This initial validity test indicated that the eHLS-Web3.0 was likely to be valuable for the characterization and understanding of EHL.

### Study 1

An effective eHealth literacy scale was developed in this study. When generating the item pool, it was hypothesized that the eHLS-Web3.0 would be categorized into 3 dimensions, namely Health 1.0, Health 2.0, and Health 3.0, for the items were generated based on the use experience of eHealth tools in different information technology (IT) generations. However, the result was different from what was hypothesized. Each item was applicable for evaluating the usage of eHealth tools in all the IT generations. The result revealed that current EHL is a comprehensive constellation of abilities where the 3 factors were correlated, distinguished, and combined, together influencing people’s usage of eHealth tools, irrespective of the generations of the tools. Moreover, during the development, the features of EHL were examined by EFA, which revealed that the data supported a 3-factor structure. This 3-factor structure differed from those in other concept-based EHL scales used in previous studies. For instance, the most widely used one, eHEALS, is a unidimensional tool that only focuses on individuals’ Health 1.0 abilities [3]. As an updated measurement tool, it is reasonable for eHLS-Web3.0 to be categorized into more factors. The 8-subscale Patient Readiness to Engage in Health Internet Technology [12] and 7-subscale eHLQ [15] considered the internal perceptions (eg, need, motivation, sense of safety, and anxiety) and external environment (relationship with service provider and access to the tools) along with the skills and abilities. In accordance with Norman and Skinner [3], this study defined EHL as the set of capabilities for people to properly understand all types of web-based health information and maintain their health and did not consider other internal and external factors for item generation so that the eHLS-Web3.0 could have a concise and targeted structure. It is worth mentioning that the factor structure of eHLS-Web3.0 is similar to the 3D eHLS [14]. The eHLS was developed following a thorough literature review, including functional, interactive, and critical dimensions of the eHLS. In the eHLS, the functional dimension is about understanding and calculating, the interactive one is about filtering, and the critical one is about cross-checking and evaluating applicability. No Web 2.0- or 3.0-related item was mentioned in the eHLS. Comparing the eHLS and eHLS-Web3.0 showed that the acquisition subscale in eHLS-Web3.0 was similar to the first and second dimensions in the eHLS (functional and interactive), whereas the verification part of eHLS-Web3.0 was close to the critical subscale in the eHLS. The application part is a newly developed dimension in the current EHL scale, which is consistent with daily experiences, as the application of information has become much more complicated than before. Others than apply the information to make health decisions or solve problems; individuals could also create their own health data, use the information to communicate, respond (ie, giving suggestions and advice, and responding to help seekers), socialize with others, and share and post information (ie, forward helpful information or post their own health or fitness data). Therefore, it is essential to enrich and include the abilities of information application into EHL. It was confirmed that the factorial structure of the eHLS-Web3.0 was reliable and valid based on statistics and theories, and in line with the actual situation.

### Study 2

The aim of this study was to examine the validity (concurrent, convergent, and construct), reliability (composite, internal consistency, and test-retest reliability) and measurement invariance (gender, major, and region) of eHLS-Web3.0. The construct validity was determined through CFA; the goodness-of-fit indices supported the 3D model structure in study 1 and confirmed the factorial composition of eHLS-Web3.0. The concurrent validity was supported by its high correlation with other well-established and validated measures of EHL (eHEALS) [3]. The convergent validity was confirmed by the AVE values, with values above 0.5 indicating acceptability. Regarding the reliability, the Cronbach α estimation showed adequate internal consistency reliability for eHLS-Web3.0 and its subscales. The item-total correlations were also tested, and they showed relatively high positive values, indicating that the items in eHLS-Web3.0 discriminated effectively between high- and low-performing participants. Given the limitation of the Cronbach α approach [46], the composite reliability coefficients were also calculated for eHLS-Web3.0, providing further positive evidence for the reliability of eHLS-Web3.0. Furthermore, the measurement invariance was measured for eHLS-Web3.0 across genders, majors, and regions. The establishment of configural, metric, strong, and structural invariances demonstrated that eHLS-Web3.0 is an appropriate and a meaningful instrument to measure EHL across diverse groups and perform comparisons. Compared with previous studies evaluating EHL among Chinese people [17,35,47], the current research had the advantage of using student samples drawn from different regions in China and from different majors. Besides, a follow-up questionnaire was designed to check if eHLS-Web3.0 was stable over time. Most importantly, this study explored the latest usage behavior related to eHealth, based on which new factors were meticulously developed to enrich the contents of EHL.

### Limitations, Strengths, and Future Directions

The study has several limitations. First, the generalizability of the findings might be hindered by the snowball sampling. As a result, a stratified sampling approach is desirable in future [48]. In addition, the results are only based on the responses provided by Chinese college students; therefore, the applicability of this tool in other groups needs to be examined. Although a purposive sample was sought, it is possible that those who responded were more interested in eHealth than nonrespondents, and this may have biased the result and the level of EHL. Moreover, self-reporting questionnaires were used, which may result in unreliability and inaccuracy because of the inherent drawbacks of self-reporting (ie, recall bias, over- or under-reporting, and social desirability) [49]. Finally, the nonhealth-related major participants were over-represented in the sample, which reflected certain realities and may also lead to some bias. Consequently, further invariance tests across majors are required with acceptably equalized sample sizes in different groups.

Despite the limitations mentioned above, the current study fills a gap in the literature, as no specific measure of EHL considering the Web 3.0 IT environment has been previously developed. A comprehensive understanding of EHL was achieved in the context of new IT and Chinese culture. By developing this reliable and valid measurement tool, this study provides an up-to-date tool to measure the level of EHL among Chinese college students and evaluate the efficacy of EHL interventions in future experimental studies on EHL. Moreover, the relationships among EHL and other health-related variables could be explored using this new tool. Apart from the previously stated theoretical values, the newly developed EHL scale may also be applied to eHealth-related interventions, such as providing new and valuable information for constructing EHL training methods.

### Conclusions

A new 24-item instrument for measuring eHealth literacy termed the eHLS-Web3.0 was developed and initially validated in this study*.* Three factors were identified in this tool, namely acquisition, verification, and application. This study validated the eHLS-Web3.0 in terms of its construct validity, convergent validity, concurrent validity, internal consistency reliability, and test-retest reliability in Chinese college students. The measurement invariance of the eHLS-Web3.0 was also confirmed across genders, majors, and regions. The 24-item eHLS-Web3.0 is a valid and reliable instrument for assessing eHealth literacy in the Chinese context.

## Appendix

Multimedia Appendix 1 Final items of eHealth Literacy Scale-Web3.0.

Multimedia Appendix 2 Results of factor loading for the construct validity test of eHealth Literacy Scale-Web3.0.

## Abbreviations

AVE: average variance extracted
CFA: confirmatory factor analysis
CFI: comparative fit index
EFA: exploratory factor analysis
EHL: eHealth literacy
eHEALS: eHealth Literacy Scale
eHLA: Health Literacy Assessment Toolkit
eHLS: electronic Health Literacy Scale
eHLQ: eHealth Literacy Questionnaire
I-CVI: content validity index of each item
IT: information technology
RMSEA: root mean square error of approximation
SRMR: standardized root mean residual
