# Supplementary material for: Measuring a Broad Spectrum of eHealth Skills in the Web 3.0 Context Using an eHealth Literacy Scale: Development and Validation Study
Source: J Med Internet Res. 2021 Sep 23;23(9):e31627. doi: 10.2196/31627 (PMC8498898; doi:10.2196/31627)
Supplement: Multimedia Appendix 1 [file jmir_v23i9e31627_app1.pdf]

Appendix 1 (a) The final items of eHealth Literacy Scale in Web 3.0 context (eHLS-Web3.0) (English version)

Items	Highly disagree	Disagree	Neutral	Agree	Highly agree
1. I know which kind of eHealth tool I should choose to fit my health needs (i.e., checking drug description, seeking health advice or making weight loss plan).	1	2	3	4	5
2. I can judge whether the eHealth tool is credible or not.	1	2	3	4	5
3. I will obtain the health information (i.e., information about medical, sport or daily care) online	1	2	3	4	5
4. I know where to find useful health resources on the Internet.	1	2	3	4	5
5. When communicating with others online, I can articulate my health-related concerns clearly.	1	2	3	4	5
6. When replying to others' health-related help-seeking online, I can provide responsible response (which means my answer can neither mislead others, nor compromise my own information security).	1	2	3	4	5
7. I can judge whether the health information online has a commercial interest (i.e., the person providing the information is for the sale of a product).	1	2	3	4	5
8. When using the eHealth tools, I will protect the originality of the information (i.e., never plagiarize others' original content, report an offence to those infringing ones).	1	2	3	4	5
9. When searching the health information online, I will check the credentials and affiliations of author.	1	2	3	4	5

10. When searching the health information online, I will check who owns the website.	1	2	3	4	5
11. When searching the health information online, I will check the date of its last update.	1	2	3	4	5
12. When searching the health information online, I will check whether other print or web resources had confirmed this information.	1	2	3	4	5
13. I know how to verify the eHealth information from multiple sources.	1	2	3	4	5
14. Even if the health information I obtained is from someone I trust, I will still check it online.	1	2	3	4	5
15. I know how to use the eHealth tools to record my health behaviors.	1	2	3	4	5
16. I know how to make use of the records on the eHealth tools to provide reference for my daily health management.	1	2	3	4	5
17. I know how to use the eHealth tools to track my health behaviors (i.e., acquainting my exercise frequency or the change curve of body fat rate).	1	2	3	4	5
18. If need, I can continuously use a certain eHealth tool (i.e., APP, intelligent body fat scale, fitness bracelet) for a long time.	1	2	3	4	5
19. If need, I can use the eHealth tools with a clear plan.	1	2	3	4	5
20. I can adjust my frequency, strength and usage pattern timely when using the eHealth tool according to the actual condition.	1	2	3	4	5
21. I know how to use the eHealth tools to post and share my eHealth behaviors (i.e., post my motion trails on health Apps or Moments on WeChat).	1	2	3	4	5
22. I know how to use the sports functions on social network services (such as WeRun on WeChat) to interact with others (e.g. thumb up,	1	2	3	4	5

forward, etc.).					
23. I will target the advanced players I follow on the eHealth tools, learn from them and catch up with them.	1	2	3	4	5
24. I will try out some health-related suggestions online and control the risks (i.e., get injured or mistake medicine).	1	2	3	4	5

Appendix 1 (b) The final items of eHealth Literacy Scale in Web 3.0 context (eHLS-Web3.0) (Chinese version)

量表条目	非常不同意	不同意	一般	同意	非常同意
1. 我知道应该选择哪种网络健康工具满足我的健康需求（如查阅药物说明、进行健康咨询、制定减肥计划等不同方面的健康需求）。	1	2	3	4	5
2. 我能够判断网络健康工具是否可信。	1	2	3	4	5
3. 我会去通过网络获取健康信息（如：医疗信息，运动保健信息等）。	1	2	3	4	5
4. 我会去通过网络获取健康信息（如：医疗信息，运动保健信息等）。	1	2	3	4	5
5. 在网上与他人交流时，我能够十分清晰地表达与健康相关的担忧。	1	2	3	4	5
6. 回答网络平台上的健康求助时，我能够给出负责任的回答（即，我的回答不会误导他人，也不会伤害自己的信息安全)。	1	2	3	4	5
7. 我能够判断网上的信息是否带有商业利益（如：提供该信息的人是为了销售某一产品)。	1	2	3	4	5
8. 使用网络健康工具时，我会去维护信息的原创性（如：不盗用他人发布的原创内容，对剽窃行为进行举报等)。	1	2	3	4	5
9. 使用网络查询健康信息时，我会去查看作者的资历证书与所属机构。	1	2	3	4	5
10. 使用网络查询健康信息时，我会去查看网页的拥有者。	1	2	3	4	5
11. 使用网络查询健康信息时，我会	1	2	3	4	5

	去查看网站最近一次的更新日期。				
12.	使用网络查询健康信息时，我会去查看是否有其他出版物或网络资源确认过此信息。	1	2	3	4	5
13.	我知道如何从多个渠道查看网络健康信息。	1	2	3	4	5
14.	即使是信任的人告诉我的健康信息，我也会上网去查阅它。	1	2	3	4	5
15.	我知道如何使用网络健康工具对我的健康行为进行记录。	1	2	3	4	5
16.	我知道如何利用网络健康工具上的记录为我的日常健康管理提供参考。	1	2	3	4	5
17.	我知道如何利用网络健康工具对我的健康行为进行追踪（如：从以往的使用记录中了解自身运动频率、体脂率等身体指标的变化曲线）。	1	2	3	4	5
18.	若有需要，我能够坚持使用某一网络健康工具（如APP，体脂秤或手环等）。	1	2	3	4	5
19.	若有需要，我能够有计划地使用网络健康工具。	1	2	3	4	5
20.	使用网络健康工具的过程中，我能够根据实际情况调整自己的使用频率、强度与方式。	1	2	3	4	5
21.	我知道如何在网络健康工具上发布和分享自己的健康行为（如：将运动轨迹分享到健康APP或朋友圈）。	1	2	3	4	5
22.	我知道如何使用社交软件上的运动功能（如微信运动）与他人进行互动（如点赞、助力等）。	1	2	3	4	5
23.	我会将我在网络健康工具上所关注的人作为目标，去效仿和赶超他（她）。	1	2	3	4	5
24.	我会去尝试网上与健康有关的提议，并控制其中的风险（如:受伤或错服药物）。	1	2	3	4	5
