# Supplementary material for: Measuring a Broad Spectrum of eHealth Skills in the Web 3.0 Context Using an eHealth Literacy Scale: Development and Validation Study
Source: J Med Internet Res. 2021 Sep 23;23(9):e31627. doi: 10.2196/31627 (PMC8498898; doi:10.2196/31627)
Supplement: Multimedia Appendix 2 [file jmir_v23i9e31627_app2.pdf]

Appendix 2 Result of factor loading for construct validity test of eHLS-Web 3.0

Items	Factor loadings	Item-total correlation
**Factor 1**		
**Item 15**	0.871	0.848
**Item 16**	0.863	0.842
**Item 17**	0.897	0.858
**Item 18**	0.858	0.787
**Item 19**	0.877	0.825
**Item 20**	0.885	0.840
**Item 21**	0.784	0.744
**Item 22**	0.785	0.757
**Item 23**	0.679	0.676
**Item 24**	0.773	0.771
**Factor 2**		
**Item 05**	0.789	0.826
**Item 06**	0.881	0.778
**Item 07**	0.864	0.747
**Item 08**	0.868	0.768
**Item 09**	0.873	0.757
**Item 10**	0.742	0.752
**Factor 3**		
**Item 01**	0.784	0.769
**Item 02**	0.820	0.793
**Item 03**	0.792	0.751
**Item 04**	0.837	0.811
**Item 11**	0.804	0.774
**Item 12**	0.841	0.822
**Item 13**	0.810	0.790
**Item 14**	0.769	0.751
**F1 with F2**	0.822	-
**F1 with F3**	0.922	-
**F2 with F3**	0.889	-
